# Synthesis of AMPSA Polymeric Derivatives Monitored by Electrical Conductivity and Evaluation of Thermosensitive Properties of Resulting Microspheres

**DOI:** 10.3390/molecules24061164

**Published:** 2019-03-23

**Authors:** Agnieszka Gola, Magdalena Sacharczuk, Witold Musiał

**Affiliations:** Department of Physical Chemistry, Pharmaceutical Faculty, Wroclaw Medical University Borowska 211, 50-556 Wroclaw, Poland; agnieszka.gola@umed.wroc.pl (A.G.); sacharczuk.magda@gmail.com (M.S.)

**Keywords:** microparticles, *N*-isopropylacrylamide, 2-acrylamido-2-methyl-1-propanesulfonic acid, volume phase transition temperature, cationic initiator, conductivity measurements

## Abstract

Four stimuli-responsive polymers of *N*-isopropylacrylamide (NIPA) and 2-acrylamido-2-methyl-1-propanesulfonic acid (AMPSA) and crosslinked derivatives by *N,N′*-methylene bisacrylamide (MBA) were synthesized: PNA, PAMPSA, PNAM, PAMPSAM. The effect of the cross-linker and methyl sulphonic acid (-CH_3_-SO_3_H) group on particle size, aggregation and volume phase transition temperature (VPTT) was investigated. Polymeric particles were synthesized via the surfactant free precipitation polymerization (SFPP) at 70 °C in the presence of cationic initiator 2,2′-azobis[2-methylpropionamidine] dihydrochloride (AMP) system. Chemical composition and morphology of investigated samples were evaluated using ATR-FTIR spectroscopy, ^1^H-NMR spectrometry and SEM-EDS techniques. The hydrodynamic diameters (HD), zeta potential (ZP), and polydispersity index (PDI) in aqueous dispersions were assessed by dynamic light scattering (DLS) between 18–42 °C. HD values at 18 °C for PNA, PAMPSA, PNAM, PAMPSAM polymers were approx. 32, 730, 715, 665 nm, and ZP values were −1.36, −0.01, 8.90, −0.09 mV, respectively. The VPTT range was observed between 29 and 41 °C. PDI’s for PNA and PNAM were low and varied between 0.276 and 0.460, and between 0.119 and 0.056, respectively. PAMPSA and PAMPSAM were characterized by higher PDI in the range 0.728–0.959 and 0.658–0.836, respectively. The results confirmed the thermal sensitivity of the synthesized polymers and indicated a significant polydispersity and aggregation tendency of the resulting molecules. The conductivity results were applied for the interpretation of the polymerization process.

## 1. Introduction

“Stimuli-responsive polymers”, among numerous micropolymers, have received considerable attention from the chemical and pharmaceutical industry. This interest results from their characteristic features, like the ability to change physical or chemical properties under the influence of various external factors: temperature, pH, UV-VIS radiation, biological factors and others; the changes are reversible after the stimulus stops.

Nowadays, the most widely studied group of “smart” micropolymers are compounds sensitive to temperature changes. The high popularity of the mentioned polymers is related to the possibility of modification of their phase transition temperature via relatively simple modifications of the synthesis process, including addition of specific co-monomers; the thermosensitivity is maintained under both “in-vitro” and “in-vivo” conditions [1,2]. The first reports about potential applications of thermosensitive polymers appeared in the 80’s in publications by Pelton and Chibante, who described the synthesis and properties of *N*-isopropylacrylamide polymer (p-NIPA) [3,4]. Since then, studies on thermo-sensitive polymer molecules have been developed very dynamically.

The mechanism of action of thermally sensitive polymers may be based on the lower critical solubility temperature (LCST)—a parameter characterizing polymers and affecting the possibility of their practical application [5,6].

The thermosensitive micropolymers have found a wide field of application in pharmacy [7] and medicine [8], mainly as potential drug carriers to ensure safe drug transport, selective release in the place of the preferred action of the drug, and decreased toxicity of the preparation to human tissues—the toxicity decrease results from drug targeting to respective tissue and thus from the decreased systemic levels of the drug in circulation [9,10,11,12,13,14]. Many groups of drugs can be delivered to the body using polymers, including anti-inflammatory drugs [15], glucocorticosteroids [16,17] and anticancer drugs [18,19,20]. The use of thermosensitive microspheres as carriers of anticancer drugs is based on the hyperthermia (1–2 °C) of tumor cells, as compared to healthy tissues. This enables the design of polymers sensitive to increased temperature, what may result in selective release of drugs into the tumor site [18]. During the drug delivery into the tissues, the EM waves may be used at the tumor site to stimulate the drug release [21]. According to the results of preclinical tests, the amounts of drug released into the tumor can be increased by using thermosensitive polymers. Thus, the cytotoxicity to cancer cells may be increased, with parallel reduction of toxicity to healthy cells [22,23,24,25,26].

The disadvantage of polymeric carriers is low efficiency of drug encapsulation in polymeric particles [27]; however, polymeric carriers with positive zeta potential may enhance the load rate, particularly in the case of small molecules of anionic drugs. Molina et al. [28] proved in an in-vitro study that for the positively charged polymer, the process of drug encapsulation into the micelle gains better efficiency, as compared to the macromolecules with negative zeta potential.

Similarly often studied, reviewed and discussed are polymers that change properties according to the pH modification. These polymers may be applied throughout the gastrointestinal tract and can be carriers of molecules of various sizes [29]. In this group of polymers, the main chain or the side chains possess ionizing functional groups. The electrostatic interactions between the polymer chains are regulated, leading to its collapse or development in solution [30,31]. The type of the functional groups in the polymer chain determines the acidic or basic nature of the polymer. Synthesis of anionic polymers is most often carried out using acrylic derivatives: acrylic and methacrylic acid [32] and sulfonamides [33]. The cationic polymers are obtained in the synthesis with respective monomers: ethyleneimine, 4-vinylpyridine, methacrylate, dimethylaminoethyl and others [34].

The aim of the research was the synthesis of cationic polymers of *N*-isopropylacrylamide (NIPA) and of 2-acrylamid-2-methyl-1-propanesulfonic acid (AMPSA) and evaluation of their physicochemical properties for use as potential carriers of biologically active molecules in targeted therapy stimulated by local increase in temperature. The AMPSA with anionic functional group was applied to evaluate the possibility of synthesis of thermosensitive polymers, potentially applied to bind cationic drugs. The cross-linker may influence the structure and physical stability of the prepared particles, and thus was applied in the study. The thermosensitive properties of PAMPSA were compared to classical PNIPA as an important factor which may influence the controlled entrapment and targeted release of drugs. Furthermore the “in situ” measurements of the conductivity of reacting mixture in the course of the synthesis were evaluated as a parameter for determination of the termination of polymerization.

## 2. Results

### 2.1. Synthesis

**Figure 1:**
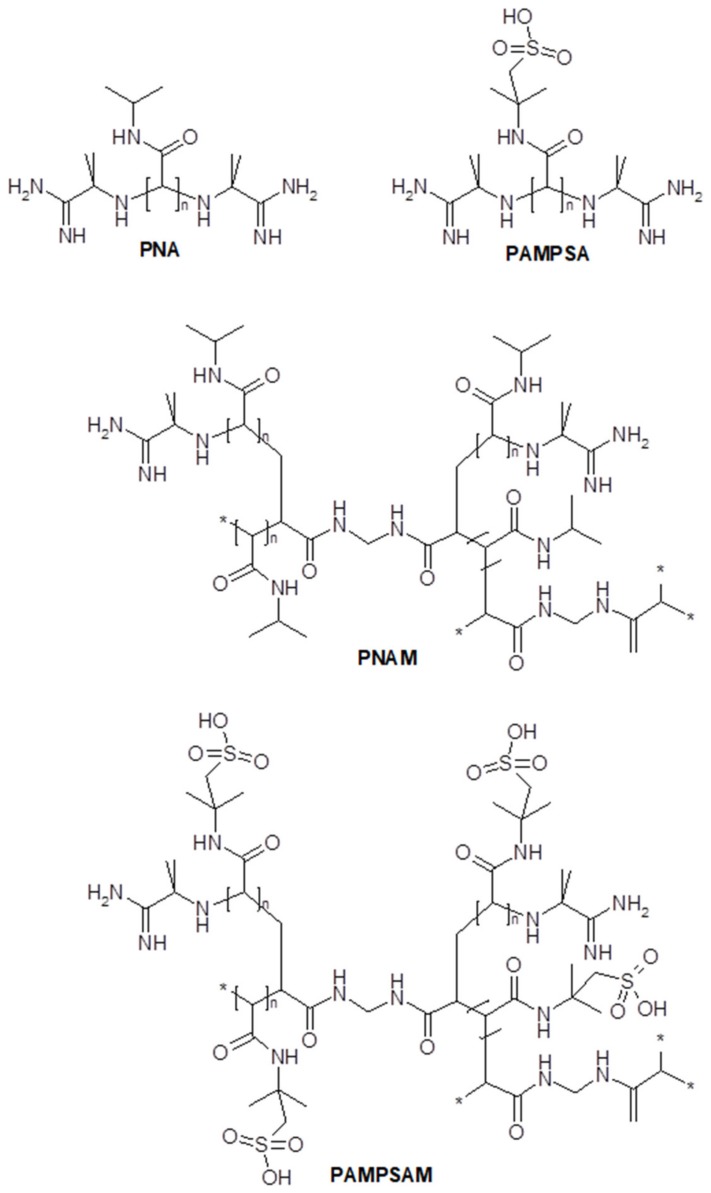
Proposed structures of synthesized polymers PNA, PAMPSA, PNAM, PAMPSAM.

In the experimental design, four types of polymeric microparticles, *N*-isopropylacrylamide (NIPA) derivatives, with cationic initiator 2,2′-azobis [2-methylpropionamidine] dihydrochloride (AMP) were synthesized: PNA, PAMPSA, PNAM, PAMPSAM (Table 1). The polymerization reactions were performed under recurrent experimental conditions, whereas the initiator concentration was adapted to the 1:0.1 molar ratio between monomer and radical. A detailed description of the synthesis is presented in Section 4, entitled Materials and methods in Section 4.2 Synthesis. The AMP concentration used in the reactions with monomer NIPA was ca. 2.24 M·dm^−3^ and in reactions with monomer 2-acrylamido-2-methyl-1-propanesulfonic acid (AMPSA) was ca. 1.24 M·dm^−3^. The merge of the components was followed by clouding of the reaction mixtures after a few minutes of the process in the case of PNA and PNAM. The turbidity initially increased and persisted until the end of the procedure. The PNA systems regained transparency after cooling. The PAMPSA and PAMPSAM systems remained transparent throughout the entire procedure. Proposed molecular structures of synthesized polymers are presented in Figure 1.

### 2.2. Conductivity Measurement Analysis

**Figure 2:**
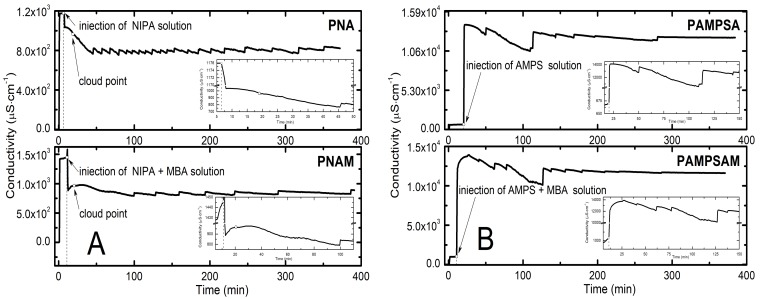
Changes in conductivity in the course of the polymerization reaction of PNA, PNAM (**A**), and PAMPSA, and PAMPSAM (**B**) in reaction mixtures.

During the synthesis, the conductivity measurement of reaction mixtures was carried out by the conductometric method at 30-s intervals. The course of conductivity changes of aqueous dispersion of PNA, PAMPSA, PNAM, PAMPSAM polymers as a function of time is shown in Figure 2A,B. In every synthesis, the first rapid jump in conductivity values occurred after adding to the reaction vessel the solution of the initiator. The value of the conductivity changed from ca. 8 μS·cm^−1^ to ca. 1200 μS·cm^−1^ and 1400 μS·cm^−1^, respectively, for PNA and PNAM and to ca. 600 μS·cm^−1^ and 900 μS·cm^−1^ for PAMPSA and PAMPSAM. In the case of the synthesis of PAMPSA and PAMPSAM polymers, the conductivity value was ca. 50% lower than for the synthesis of PNA and PNAM polymers. Subsequently, the system was left for a few minutes to equilibrate the initiator molecules in water; in this period the conductivity value remained stable. Next, a very rapid and short change in conductivity occurred after injecting the aqueous solution with the monomer solution. In the reaction system of PNA and PNAM, the conductivity decreased, whereas in the case of PAMPSA and PAMPSAM, polymerization conductivity increased. In the next stage, in each system the conductivity value gradually decreased.

According to the data in Figure 2A-top in the PNA system, during ca. 30 min, the values of conductivity changed from 1170 μS·cm^−1^ to ca. 760 μS·cm^−1^ and stayed at this level with small fluctuations until the end of the polymerization process. Values of the conductivity of the PNAM system, after addition to the reaction vessel the mixture of the monomer with cross-linking agent, for fifteen minutes, increased from 886 to ca. 980 μS·cm^−1^, followed by a slow 60-min decrease to ca. 800 μS·cm^−1^. The achieved value of conductivity remained unchanged until the end of the synthesis (cf. Figure 2A-bottom). In both PNA and PNAM systems, respectively, after ca. 10 and 8 min from the injection of the monomer mixture, turbidity was observed. The values of conductivity recorded at that time were ca. 964 μS·cm^−1^ for PNA and 967 μS·cm^−1^ for PNAM systems.

After the monomer addition, the measured conductivity values for the PAMPSA system varied, within 21 min, between ca. 13,820 μS·cm^−1^ and ca. 12,700 μS·cm^−1^. This value range with slight deviations resulting from the accumulation of gas in the conductivity cell, was maintained until the end of the measurement.

In the case of the PAMPSAM system, the very rapid increase was followed by 10 min of a slight increase phase; the conductivity increased from ca. 12,250 μS·cm^−1^ to ca. 13,680 μS·cm^−1^. Subsequently, the conductivity decreased and after ca. 120 min, stabilized at the level of about 12,000 μS·cm^−1^.

### 2.3. Attenuated Total Reflection Fourier Transform Infrared Spectroscopy Analysis (ATR-FTIR)

**Figure 3:**
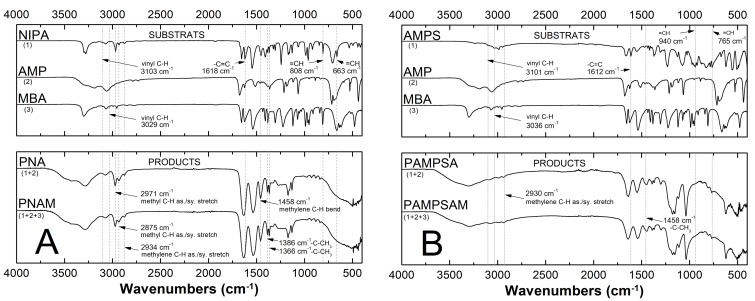
Fourier transformed infrared spectroscopy with attenuated total reflectance (ATR-FTIR) spectra of monomers: *N*-isopropylacrylamide—NIPA (**A**-Top) and 2-acrylamido-2-methyl-1-propanesulfonic acid —AMPS (**B**-Top), initiator: 2,2′-Azobis(2-methylpropionamidine) dihydrochloride—AMP (**A**, **B**-Top), crosslinker: *N*,*N′*-methylene bisacrylamide—MBA—(**A**,**B**-Top); and synthesized polymers: PNA, PNAM (**A**-Bottom) and PAMPSA, PAMPSAM (**B**-Bottom).

The attenuated total reflection Fourier transform infrared spectroscopy ATR-FTIR spectra of monomers NIPA, AMPS, initiator AMP and cross-linker MBA were compared to the spectra of resulting products PNA, PAMPSA, PNAM, and PAMPSAM (cf. Figure 3A,B), to confirm that polymerization occurred. The expected and characteristic bands of functional groups for NIPA, AMP, MBA are shown in the upper part of the charts in Figure 3A,B. PNA and PNAM spectra are presented in the bottom part of the charts in Figure 3A,B.

### 2.4. Nuclear Magnetic Resonance Spectroscopy Analysis (^1^H-NMR)

**Figure 4:**
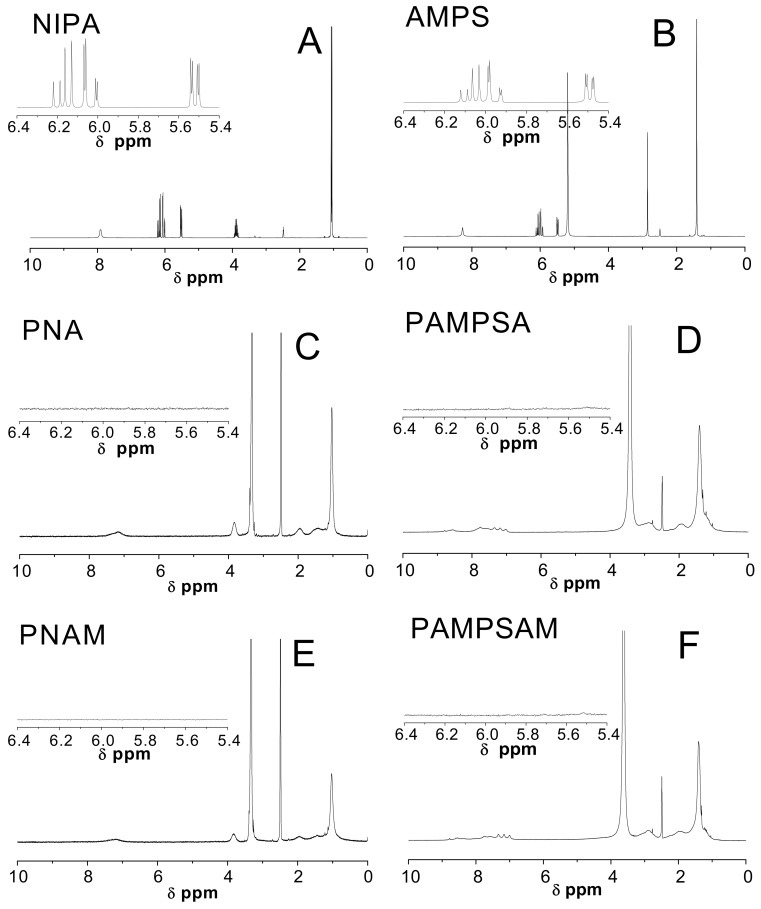
^1^H-NMR spectra of monomers: NIPA-(**A**) and AMPS-(**B**) and four synthesized polymers PNA-(**C**); PAMPSA-(**D**); PNAM-(**E**); PAMPSAM-(**F**); The expanded areas in the ^1^H-NMR spectra present the resonance range of the vinyl protons.

The structure and composition of prepared polymers PNA, PAMPSA, PNAM, and PAMPSAM were also supported by ^1^H-NMR spectroscopy (Figure 4). In order to explore whether the polymerization had taken place, the ^1^H-NMR spectra of synthesized polymers with spectra of monomers NIPA and AMPS in DMSO-*d*_6_ solutions were compared.

### 2.5. Hydrodynamic Diameter (HD)

**Figure 5:**
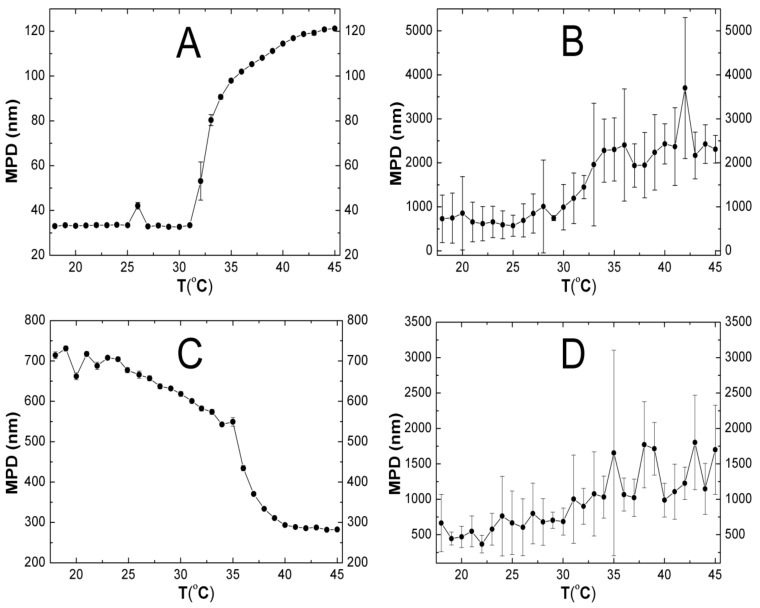
**A**–**D**. The effect of the temperature on hydrodynamic diameter of the prepared particles PNA-(**A**), PAMPSA-(**B**), PNAM-(**C**), PAMPSAM-(the **D**), MPD—mean particle diameter.

Figure 5A–D presents the influence of the temperature on the hydrodynamic diameter of the four synthesized polymers PNA, PAMPSA, PNAM, PAMPSAM in water suspensions. The hydrodynamic diameter of the resulting polymers, PNA, PAMPSA, PNAM, PAMPSAM, at 18 °C was approximately 32, 730, 715, and 665 nm, respectively. As illustrated in Figure 5A,B the size of polymers PNA and PAMPSA **remained** with small deviations, constant until the temperature of ca. 31 °C. Above these temperature, up to 45 °C, the hydrodynamic diameters clearly start to increase, to 120 nm for PNA and to 2200 nm for PAMPSA. In the case of crosslinked polymer PNAM (cf. Figure 5C), as the temperature increased, the hydrodynamic diameter gradually decreased from 730 nm at 18 °C to 550 nm at 35 °C. In the range of 35–40 °C, a rapid reduction in the hydrodynamic diameter of the polymer to approx. 290 nm was observed. This value, with slight deviations, remained essentially constant until the end of the assay. Figure 5C shows classical dependence of hydrodynamic diameter on temperature with a typical sharp inclination at 36 °C, indicating the temperature of phase transition. Above these temperatures, as predicted, particles collapsed and the hydrodynamic diameter decreased. In Figure 5D, it is not possible to clearly indicate the phase transition temperature. There is no sharp change in hydrodynamic diameter evolution versus temperature, as is commonly observed in the graph for D_H_(T). Nevertheless, a relationship between the size and the temperature can be observed. As the temperature rised from 18 °C to 45 °C, the hydrodynamic diameter increased from 665 nm to 1700 nm.

### 2.6. Polydispersity Index (PDI)

**Figure 6:**
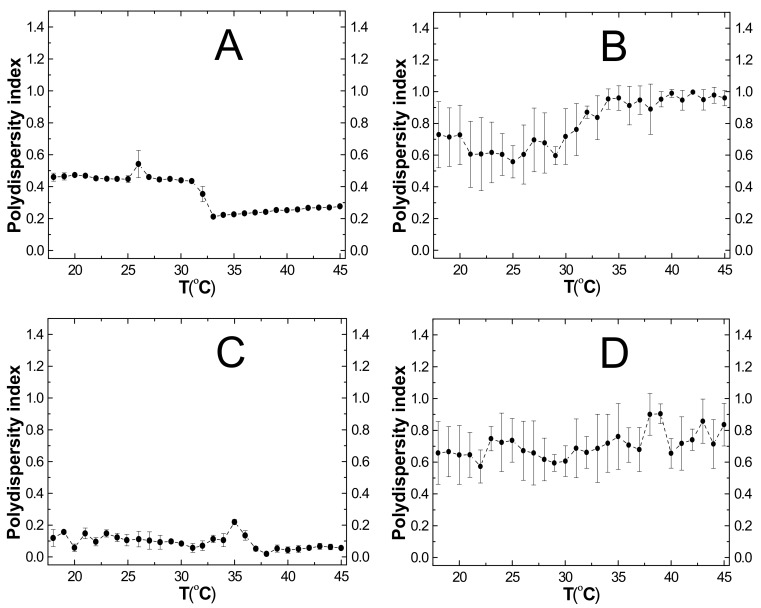
**A**–**D**. The effect of temperature on the polydispersity index (PDI) of the prepared microparticles PNA-(**A**), PAMPSA-(**B**), PNAM-(**C**), PAMPSAM-(**D**) obtained from dynamic light scattering (DLS) measurements.

Conventional radical polymerization usually results in a nonhomogeneous batch of polymers of various sizes and molecular weights. Figure 6A–D depicts changes in the polydispersity of synthesized polymer molecules as a function of temperature. The polydispersity index values of the obtained polymers in the temperature range 18–45 °C showed relatively low values not exceeding unity. As shown in Figure 6A–D, the PNA and PNAM polymers clearly show lower values of PDI than polymers PAMPSA and PAMPSAM. Changes in the polydispersity index for PNA and PNAM polymers ranged from 0.276 to 0.460 and 0.119 to 0.056, respectively, accompanied by small errors bars representing the standard deviation of the five measurements for one temperature. In addition, in the diagrams in Figure 6A,C, a singular notable decrease of PDI values was observed. Significant changes in PDI values from ca. 0.434 to 0.212 occurred around 31 °C and from ca. 0.219 to 0.019 around 35 °C for PNA and PNAM, respectively. For polymer PAMPSA, the PDI values changed from 0.728 to 0.959 (cf. Figure 6B) and for polymer PAMPSAM, from 0.658 to 0.836 (cf. Figure 6D). Values of PDI for PAMPSA and PAMPSAM polymers in the whole measurement temperature range 18–45 °C remained more or less constant level without any pronounced and characteristic leap. Changes in PDI factor for PAMPSA and PAMPSAM polymers occurred with small fluctuations and were within the limits of measurement error.

### 2.7. Zeta Potential (ZP)

The zeta potential, defined as the electric potential measured on the hydrodynamic shear plane, is an important parameter of stability of colloids or microparticles in suspension. Zeta potential measurement for synthesized polymers was carried out at four temperatures: 18, 25, 32 and 45 °C in non-buffered, aqueous solution. The data are presented in the Table 2.

All synthesized polymers exhibited an increase of the electrophoretic mobility when the temperature increased from 18 to 45 °C. The less significant temperature dependence of zeta potential is indicated by polymers PAMPSA and PAMPSAM. Positive values of zeta potential in the entire assessed temperature range were found only for the PNAM polymer. In other cases, positive zeta potential appeared at 32 and 45 °C for PNA, and at 25 and 45 °C for PAMPSA.

### 2.8. Scanning Electron Microscopy with Energy Dispersive Spectroscopy System (SEM-EDS) Analysis

**Figure 7:**
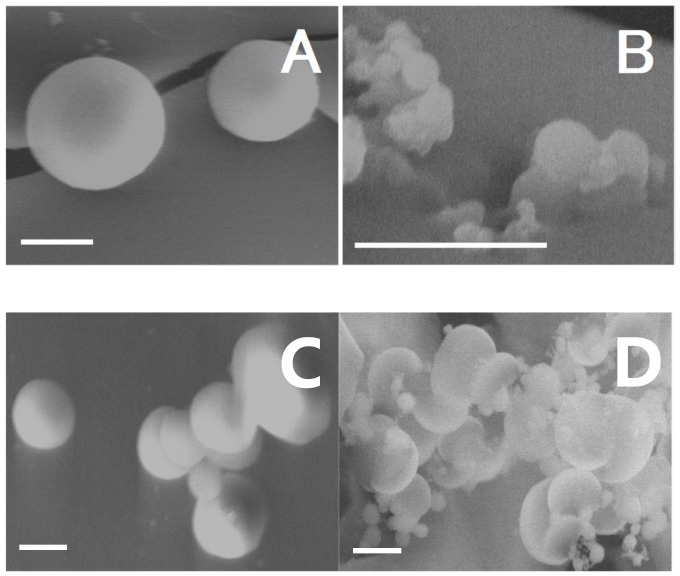
**A**–**D**. Scanning electron images of the selected polymeric particles PNA-(**A**), PAMPSA-(**B**), PNAM-(**C**), PAMPSAM-(**D**). The bar represents 2 µm.

The scanning electron microscopy method was used to determine morphology and also to study the size of synthesized polymers. A few representative SEM micrographs of PNA, PAMPSA, PNAM, PAMPSAM are depicted in Figure 7A–D. The results of SEM show that all the polymers, PNA, PAMPSA, PNAM, PAMPSAM, synthesized via suspension polymerization technique, had a spherical shape. Most of the synthesized PNA and PNAM polymeric spheres had diameters of about 2 µm. The particle diameters of spherical PAMPSA and PAMPSAM were very diverse and they ranged from about 0.06 to 0.6 µm and from about 0.25 to 2.5 µm, respectively.

**Figure 8:**
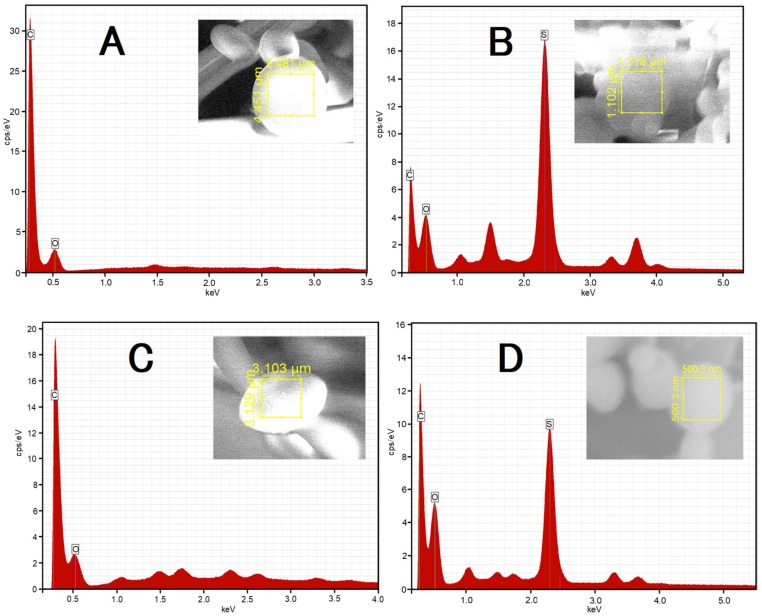
**A**–**D**. Energy dispersion spectrum of prepared polymer particles PNA-(**A**), PAMPSA-(**B**), PNAM-(**C**), PAMPSAM-(**D**) in the inserted area marked in corresponding SEM micrographs.

The identification and confirmation of the type of atoms included in spherical forms of synthesized polymers of individual particles was examined using the Energy Dispersive X-Ray Spectroscopy within the SEM device (EDS). The qualitative and semi-quantitative determination of the elemental composition was made using surface analysis of the tested material on the given field area. The EDS method is closely related to the morphology of the sample and the conversion of the intensity of peaks from energy dispersion diagrams into the percentage of elements; in non-standard analysis, should be treated as an approximate assessment and the quantitative value should be considered semi-quantitative and used for illustration. The obtained EDS spectra with inserted SEM micrographs of reference samples are presented in Figure 8A–D. The EDS investigations of PNA, PNAM, PAMPSAM, PAMPSA samples, as predicted, revealed the concentration of carbon, as compared to oxygen in each sample (cf. Figure 8A–D). Additionally on the EDS spectrum of PAMPSAM and PAMPSA, samples with a high concentration of sulfur were detected. Furthermore, with respect to the expected elements, the EDS analysis revealed the presence of trace amounts, less than 1% weight, of elements K, Ca, Al, Si, Cu, Cl, Na. The presence of these elements may indicate contamination of the samples during preparation for measurement.

## 3. Discussion

### 3.1. Synthesis

In the course of the synthesis, high turbidity was observed in the case of PNA and PNA polymers, confirming the distinctive VPTT macroscopically.

### 3.2. Conductivity

Conductivity measurements, due to their simplicity and accuracy, have been successfully used to evaluate the release of drug substances from the carrier [35]. Based on our previous research [36], this method was used to determine the stages and the end of the polymerization process. The results of conductivity measurements carried out in the course of the synthesis of PNA, PNAM, PAMPSA, PAMPSAM polymers seem to preliminarily confirm that conductivity measurements may reflect the course of free radical polymerization. The raw conductivity results presented characteristic changes in conductivity after one by one addition of synthesis substrates. The time points in which the conductivity stabilized at a constant level were also designated. The conductivity reached a constant level, after ca. 50 and ca. 100 min of the process for polymers PNA and PNAM, respectively, and after ca. 150 min of the process for PAMPSA and PAMPSAM. The detailed evaluation of the measurements may give insight into the stages of the process. The initial quick increase results from the decomposition of the initiator molecules to the radicals. The recorded high value of conductivity reflects at least the conductivity of the radicals originating from the initiator (Figure 2A,B). The consequent addition of the monomer results in the consumption of the initiator; however, the consequent conductivity level of the reactant mixture depends on the type of monomer. The addition of a unionizing monomer, i.e., NIPA, favored the decrease in conductivity—Figure 2A, whereas the ionizing monomer AMPS resulted in increased conductivity—Figure 2B. The addition of a cross-linker results in a slight increase in conductivity in both systems—Figure 2A,B, bottom parts; however the increase differs and depends on the type of applied monomer. The unionizing monomer is responsible for a rather low conductivity increase in the presence of the cross-linker, but the ionizing monomer causes a more pronounced increase in the conductivity. This type of behavior can be explained by the fact that the NIPA monomer does not dissociate and after entering to the system, reacts immediately with radicals. By contrast, the highly polar AMPS molecule in polar solvents is strongly solvated and exhibits a high dissociation ability, producing mobile sulfonate ions [37,38] resulting in a significant increase in conductivity after introduction. The cause of the observed phenomenon should be connected to ionization of nitrogen atoms in the cross-linker molecule in the case of unionizing monomer NIPA, and protonizing activity of the sulphonic group in the ionizing monomer AMPS. The further decrease of the conductivity is a consequence of elongation of the polymer chain, and clearly the ionizing AMPS retards the stabilization of the conductivity level, due to the presence of sulphonic groups, compared to the NIPA substrate. The following occurrence of a plateau may be an important informative factor, confirming the completion of the polymerization process. Limited fluctuations of the conductivity values depicted in charts in the plateau area were due to the accumulation of gas bubbles in the conductometric cell during synthesis. Moreover, measurements of the conductivity of the reaction mixture in the course of polymer synthesis and the conductivity of substrates carried out separately under the synthesis conditions enable the confirmation that the polymerization reaction took place and new molecules were formed.

### 3.3. ATR-FTIR

The following bands characteristic of unsaturated compounds disappeared in the spectra of polymers: at 1618 cm^−1^, 808 cm^−1^, 663 cm^−1^ with respect to stretching [39], out-of-plane and wagging vibrations [40] for NIPA, respectively, and at around 1612 cm^−1^, 940 cm^−1^, 765 cm^−1^ for AMPS assigned to stretching vibrations in vinyl group H–C=C, and also at 3103 cm^−1^ and 3029 cm^−1^ for NIPA [41] and at 3101cm^−1^ and 3036 cm^−1^ for AMPS related to stretching vibrations in C=C group. This is proof of the successful occurrence of polymerization and confirms the absence of unreacted monomers in samples of synthesized polymers. Additionally, on the spectra of polymers there is an increase in the intensity of peaks in the area 2930–2940 cm^−1^ and at around 1458 cm^−1^ related to -CH_2_ groups from the polymer chain. The very strong bond observed at 1637 cm^−1^ in the spectrum of PNA and PNAM and at 1643 cm^−1^ in the spectrum of PAMPSA and PAMPSAM may be the result of overlapping bands from C=O stretching and of NH_2_ stretching and deformation vibration frequencies. In the spectra of PAMPSA and PAMPSAM, the observed bands at 1038 cm^−1^ and 1179 cm^−1^ were assigned to symmetric and asymmetric stretching vibrations O=S=O of the SO_3_H group.

### 3.4. H-NMR

The ^1^H-NMR spectra of polymers PNA, PAMPSA, PNAM and PAMPSAM show that polymerization has taken place and both NIPA and AMPS monomers were completely consumed in these processes, which was evidenced by the absence of characteristic peaks of the unsaturated –C=CH group at δ 5.4–6.4 ppm (see inserts in Figure 4C–F). Moreover, all spectra of polymeric products contained strong peaks at δ 3.26–3.64 ppm of the polymer chain protons attributed to methylene groups. In the spectra of PNA and PNAM, we also identified peaks with chemical shifts around δ 7.17 ppm that belonged to the –NH proton and four other signals: at δ 3.83 ppm from single protons of the isopropyl group, at δ 1.96 ppm from methine proton, weak broad peak around δ 1.42 ppm from the methylene group and at δ 1.03 from methyl protons of the isopropyl group. In the PAMPSA and PAMPSAM spectra, the observed maxima in the range 7.00–8.79 belonged to the –NH and –NH_2_ protons, at δ 2.89 ppm and δ 2.77 ppm to methine proton, at around δ 2.00 to methylene protons connected with SO_3_H group and at δ 1.40 ppm, characteristic of methyl proton of the isopropyl group.

### 3.5. HD

Based on the DLS data, the hydrodynamic diameter of evaluated non–cross linked polymers changed with rising temperature, and the diameter of NIPA derivatives changed very dynamically. The cross-linked polymer PNAM showed the expected decrease in the hydrodynamic diameter of the molecules at increased temperature. In the case of non-crosslinked polymer PNA, a significant increase in hydrodynamic diameter above 32 °C, possibly due to the aggregation of molecules, has been demonstrated, similar to the case of polymers based on AMPS; however, their diameter increased more slowly compared to NIPA derivatives. Also, other systems applied, e.g., in microtechnology, may present specific aggregation properties, utilized for preparation of self-assembling structures [42,43]. In accordance with the DLVO [44] theory, the colloidal stability depends on the attraction of van der Waals forces, the repulsive steric forces and electrostatic forces. The van der Waals forces are conducive to aggregation and the repulsive steric forces and electrostatic forces counteract them. Below the VPTT, in aqueous suspension of polymers, the hydrogen interactions between water molecules and hydrophilic polymer groups dominate and exceed the van der Waals interactions, which leads to a decrease of attractive forces [45], and diminished aggregation. Moreover, under these conditions, the polymeric tails of the expanded polymeric net influence the colloidal stabilization as steric stabilizers. In the case of PNAM polymer, the steric stability above VPTT can be enhanced by cross-linking, even when the polymer tails collapse and do not contribute to these steric effects. The effect of the cross-linker content on the size of polymer particles at different temperatures was reported by McPhee at all [46] and confirmed by other researchers [47,48]. Increases occurred in the hydrodynamic diameter of polymers PNA, PAMPSA, PAMPSAM (Figure 5A–D) at higher temperatures, when hydrogen bonds of polymer-water are broken, and the protective stabilizing steric effects disappear, which may be the results of an increase in hydrogen binding forces between polymer particles. The hydrolysis adversely affects the stability of the formed molecules; therefore, it is important to ensure conditions in the course of the reaction to reduce these process. One of the ways is to maintain the appropriate pH of the reaction mixture. According to Ito research [49], a pH increase above 7 leads to an increase in the rate of hydrolysis. The syntheses of NIPA and AMPSA derivative polymers were carried out at pH ≈ 7 and pH ≈ 4, respectively. These conditions reduce the hydrolysis of the initiator to a minimum; however, even the minimum degree of hydrolysis may affect the stability. The increase in particle size can be the result of aggregation of the particles as a result of intermolecular interactions enhanced at a higher temperature. The comparison of charts in Figure 5A–D suggest that the NIPA derivatives are more stable than the AMPS derivatives over the entire temperature range, which is evidenced by the size measurements with errors smaller than 5%. Significant experimental errors associated with size measurements of PAMPSA and PAMPSAM polymers can result from the aggregation of particles already at 18 °C. Moreover, in the case of thermally sensitive polymers, it is expected that above the phase transition temperature, the particle size will decrease; this effect was obtained only in the case of the MBA-cross-linked NIPA polymer—PNAM. In the crosslinked molecules, the structure of polyNIPA is more stable, which prevents aggregation but increases the size of the molecule, due to the formation of a compact structure. Polymers synthesized on the basis of AMPS do not show the phase transition temperature in the tested temperature range. The visible difference between sizes obtained from DLS measurements and from demonstrative SEM observations may result from the agglomeration of the desiccated particles – similar phenomena were observed in other research, so we followed the DLS pattern in the present discussion, not the results of SEM visualizations [50].

### 3.6. PDI

Changes in the PDI of the studied polymers showed a relatively small polydispersity of PNA and PNAM polymers and slightly higher polydispersity in the case of PAMPSA and PAMPSAM polymers. The research also showed that values of PDI for polymers synthesized on the basis of NIPA decrease with increasing temperature with a characteristic point of reflection around 33 °C for PNA and around 38 °C for PNAM; the molecules were uniform as the temperature increased. Size of molecules based on AMPSA differentiated with increasing temperature, which is consistent with the results of the hydrodynamic diameter measurement [35]. The slight increase in the polydispersity value above the 33 °C temperature in the case of PAMPSA and PAMPSAM may be ascribed to aggregation of molecules.

### 3.7. ZP

Potential zeta is proportionally related to the electrophoretic mobility by the Smoluchowski equation. Low values of zeta potential result from small electrophoretic mobility, a parameter characteristic for a given colloidal system. According to Pelton et al. [4], the absolute value of the electrophoretic mobility increased with increasing temperature; at lower temperatures, electrostatic repulsive forces have less impact, which favors the process of aggregation. Polymer molecules in accordance with literature data are stable and do not aggregate when its absolute value is equal or greater 30 mV [35]. The results presented in Table 2 show that HD’s of PNA and PNAM polymers are stable at 45 °C; however, at lower temperatures they are not stable. The HD’s of PAMPSA and PAMPSAM polymers were not stable in the entire tested temperature range.

### 3.8. SEM-EDS

SEM results in Figure 7A–D demonstrate that free radical aqueous precipitation polymerization method yielded the polymeric particles of PNA, PNAM, PAMPSA and PAMPSAM in spherical shape. SEM images of PAMPSA and PAMPSAM polymers, containing sulphonic groups in their polymer chains, presented significant variation in particle size (cf. Figure 7B,D). Most of the particles were in the nano or submicron size depending on the type of substrate used in synthesis. Inhomogeneity in the PAMPSA and PAMPSAM samples is reflected in the results of DLS, PDI and ZETA analysis. The large variation in the size of the polymer spheres in the samples resulted in a significant increase in the deviation value. In addition, SEM images of PAMPSA and PAMPSAM samples confirm a much higher particle count than in PNA and PNAM samples, which suggests that sulphonic groups in the particles may drive agglomeration.

EDS analysis was used to obtain information about elemental composition of the samples and also to estimate the weight percentage of elements in the samples. X-ray microanalysis confirmed the presence of carbon as the main component in PNA sample in the amount 78.56 ± 7.91 and showed similar values of % weight obtained for carbon and oxygen in samples of PAMPSA (C: 27.30 ± 1.08, O: 30.42 ± 1.50) and PAMPSAM (C: 41.37 ± 1.95, O: 43.85 ± 2.58) and for carbon and nitrogen in the sample of PNAM (C: 40.35 ± 3.69, N: 39.38 ± 4.32). Contrary to expectations, the presence of nitrogen was found only in the PNAM sample; however, the absence of a signal from nitrogen in other EDS spectra of PNA, PAMPSA and PAMPSAM samples may result from a smaller share of nitrogen in the scanning area or due to the jamming of its signal by other elements occurring in a significant amount such as carbon or sulfur. Unfortunately, this can interfere with the overall weight ratio of the other components in samples. According to the graphs shown in Figure 8B,D, the presence of sulfur from the sulfonic group in the AMPS monomer is clearly visible. Obtained values of weight percent for sulfur in PAMPSA and PAMPSAM samples are 27.54 ± 0.90 and 10.56 ± 0.42, respectively. Sensitivity of the EDS method is low for elements with low atomic numbers, lower than Z = 3; therefore, no hydrogen on the EDS spectra can be seen. Summarizing the results obtained from the semi-quantitative EDS analysis can be convincing proof of the content of the monomer used in the obtained polymer but cannot provide information about, e.g., chemical bonds or chemicals states.

## 4. Materials and Methods

### 4.1. Materials

*N*-isopropylacrylamide (NIPA, 99%, Sigma-Aldrich Chemical, St. Louis, MO, USA), 2-acrylamido-2-methyl-1-propanesulfonic acid (AMPS, 99%, Sigma-Aldrich Co., St. Louis, MO, USA), *N*,*N*′-methylene bisacrylamide (MBA, 99%, Sigma-AldrichCo.,St. Louis, MO, USA), 2,2′-Azobis(2-methylpropionamidine) dihydrochloride (AMP, 97%, Sigma-Aldrich, Sternheim, Germany) were obtained from commercial and industrial suppliers and were used without further purification. Dialysis tubing cellulose membrane (MWCO 12,000–14,000 Da) was received from Sigma-Aldrich CHEMIE GmbH, Sternheim, Germany. Deionized water fulfilled requirements of PN-EN ISO 3696: 1999 for analytical laboratories; it was filtered in an HLP 20 device equipped with a microfiltration capsule 0.22 μm (Hydrolab, Straszyn, Poland) and was applied in all following procedures.

### 4.2. Synthesis

Four syntheses of polymeric microspheres (PNA, PAMPSA, PNAM, PAMPSAM) were performed. The polymers were prepared by the free radical aqueous precipitation polymerization method without the use of a surfactant (surfactant free precipitation polymerization, SFPP). The dry sample of free radical cationic starter—AMP was added directly to the 2000 mL 4-necked round bottom flask reaction vessel filled deionized water (~0.9 μS·cm^−1^). The system was heated up to 70 °C, equipped with a reflux condenser, thermometer, and conductivity cell (K = 1 cm^−1^). The solution of the initiator was stirred (250 rpm) and degassed with N_2_ for ca. 10 min before introducing the appropriate monomer (NIPA or AMPS) and, in two syntheses of four, cross-linker (MBA). The monomers and cross-linker, before injections, were dissolved in deionized water at room temperature. The total volume of the reaction mixture was 1000 mL. The polymerization was carried out at 70 °C for 6 h under nitrogen atmosphere with respective mixing. Table 1 shows the substrate composition for performed polymerizations reactions. The polymer solution was purified by a minimum of six dialysis cycles against distilled water at room temperature. During dialysis, the water was stirred and changed every 24 h. The purification procedure was completed when the conductivity of water was less than 1.5 µS cm^−1^.The samples were frozen, and lyophilized by Alpha 1–2 LD (Martin Christ Freeze Dryers, Osterode am Harz, Germany) for 32 h, and stored dry.

### 4.3. Conductivity Measurements

The conductivity changes of the aqueous phase of the reaction mixture in the course of the polymerization reaction were measured by a conductivity meter, model CC-505 (Elmetron, Zabrze, Poland) equipped with an EC-60 immersion conductivity cell (Elmetron, Zabrze, Poland). The conductivity sensor containing two platinum electrodes covered with platinum black, placed inside a glass measuring cell remained in permanent contact with reaction mixture. The range of the conductometric cell constant was K = 1.0 ± 0.2 cm^−1^. Conductivity measurements were performed at 70 °C with direct temperature control with an accuracy ±0.5 °C. Conductivity was measured as a function of time and recorded on a PC.

### 4.4. Attenuated Total Reflection Fourier Transformed Infrared Spectroscopy measurements (ATR-FTIR)

The infrared spectra of dry samples were acquired using attenuated total reflection Fourier transformed infrared spectroscopy (ATR-FTIR) in a Nicolet iS50 FT-IR spectrometer (Thermo Fisher Scientific Madison, WI, USA) with DTGS (Deuterated Triglycine Sulphate) detector. The spectra were collected over the wavenumber ranging from 4000 cm^–1^ to 400 cm^–1^ at 32 scans per sample and at a resolution of 4 cm^−1^. The reference spectra using a blank ATR were recorded and subtracted from each spectrum before data output. The ATR measurements were made by uniform compression of a small amount of compound placed directly on the area of the monolithic diamond crystal using a pressure arm. Before each measurement, the crystal surface and the tip of the pressure tower were cleaned using methanol-soaked tissue paper.

### 4.5. Nuclear Magnetic Resonance Spectroscopy measurements (^1^H-NMR)

The proton NMR spectra were recorded on a Spectrometer Bruker operated at 300 MHz (Bruker, Rheinstetten, Germany). The polymer and substrates solutions were prepared by dissolving about 10 mg of each compound in 7 mL of deuterated dimetylsulfoxide (DMSO-d6, δ = 2.49), at 26 °C.

### 4.6. Hydrodynamic diameter (HD)and polydispersity index(PDI) measurements

The analysis of the particle size of the synthesized polymers in aqueous dispersions was performed by the dynamic light scattering method (DLS). A Zeta Sizer Nano device of Malvern Instruments was used (Malvern Instruments, Malvern, UK). Measurements were carried out over the temperature range of 18–45 °C using standard-red He-Ne, 4 mW, laser light of wavelength λ = 633 nm, recorded at an angle of 173° backscattering setting. The samples were not diluted, due to the low concentrations of the purified product. The light intensity was regulated during the measurement sequence by an automatically set laser attenuator. Five measurement rounds were carried out at each temperature for hydrodynamic diameter. The number of repetitions in one round was determined automatically in the range of 10 to 100 measurements. The equilibration time for each sample after the temperature change was 120 s. The measurements of hydrodynamic diameter were carried out in polyacrylic disposable DTS-0012 cuvette (Malvern Instruments, Malvern, UK) filled by 1 mL solution of polymer obtained after dialysis cleaning. Additionally, in the course of photon correlation spectroscopy measurements, the polydispersity index (PDI) as a parameter of the particle size distribution of the microparticles was calculated, according to the device ISO standard document 13321:1996E [51].

### 4.7. Zeta Potential (ZP) Measurements

Zeta potential parameter (z) was determined from electrophoretic measurements–mobility of synthesized polymers particles in aqueous dispersion in the electric field using a Zeta Sizer Nano device of Malvern Instruments (Malvern Instruments, Malvern, UK). The speed of particles between the electrodes was measured in special U-shaped cuvettes type DTS-1070 with installed electrodes (Malvern Instruments, Malvern, UK) using the Doppler effect (Laser Doppler Velocimetry, LDV). The Smoluchowski model approximation to Henry’s equation was applied (f(Ka) = 1.5) for the calculations of zeta potential values. The influence of temperature on the zeta potential was studied in the temperature range 18–45 °C. For each temperature, three measurements rounds were performed. The equilibration time was 120 s, following every 1 °C increase in temperature.

### 4.8. Scanning Electron Microscopy With Energy Dispersive Spectroscopy System (SEM-EDS) Measurements

The morphologies of the freeze-dried samples of PNA, PAMPSA, PNAM, PAMPSAM spheres were examined using a scanning electron microscope (SEM, Quanta 650 FEG, FEI Company, Hillsboro, OR, USA) equipped with a field emission gun (FEG) which allows for bright-field and dark-field sample imaging and with a large field detector (LFD) for dry and non-conductive samples in low vacuum mode (0.8–1.5 Torr). The operating voltage was 10.0 kV, working distance (WD) 10–12 mm and spot size 3.0.X-ray; microanalysis of the tested material was done using an energy dispersive X-ray spectroscopy (EDS) analyzer (QANTAX, Bruker Nano GmbH, Berlin, Germany), a component of the scanning microscope.

## 5. Conclusions

Four polymers were synthesized: PNA and PNAM derivatives of *N*-isopropylacrylamide and PAMPSA and PAMPSAM derivatives of 2-acrylamido-2-methyl-1-propanesulfonic acid. PNAM and PAMPSAM polymers were cross-linked by methylene-bis-(acrylamide). The monitoring of the course of syntheses was performed via conductometric measurements. The polymers, purified by diffusion from the semipermeable membrane, were studied to evaluate the possibility of application as thermosensitive drug carriers. The implementation of the monomers and co-monomers into the structure of the synthesized polymers was confirmed by ATR-FTIR and ^1^H-NMR spectroscopy. Particle size vs. temperature, potential zeta and polydispersity index were determined using the DLS method. The pH of aqueous suspensions of the synthesized polymers was also measured. The results confirmed the thermal sensitivity of the synthesized polymers and indicated a significant polydispersity and aggregation tendency of the resulting molecules. The pH measurements of polymers based on 2-arylamido-2-methyl-propanesulfonic acid indicate that they could find use as drug carriers for dermal application.

## Figures and Tables

**Table 1:** Compositions of PNA, PAMPSA, PNAM, PAMPSAM.

Type of Polymer Microparticle System	Monomer [mol]		Cationic Initiator [mol]	Cross-Linker [mol]
	NIPA	AMPS	AMP	MBA
PNA	4.433 × 10^−2^	-	2.238 × 10^−3^	-
PAMPSA	-	2.453 × 10^−2^	1.232 × 10^−3^	-
PNAM	4.429 × 10^−2^	-	2.224 × 10^−3^	3.256 × 10^−3^
PAMPSAM	-	2.416 × 10^−2^	1.239 × 10^−3^	3.282 × 10^−3^

**Table 2:** Value and standard deviations of zeta potential data and pH data for synthesized microspheres PNA, PAMPSA, PNAM, PAMPSAM.

Type of Polymer Microparticles System	Zeta Potential [mV]				pH
	Measurement Temperatures [°C]				
	18	25	32	45	25
PNA	−1.36 ± 0.87	−0.34 ± 0.83	1.65 ± 0.75	47.32 ± 1.88	7.39
PAMPSA	−0.01 ± 0.37	0.15 ± 0.22	−0.23 ± 0.05	0.06 ± 0.34	4.42
PNAM	8.90 ± 0.11	10.00 ± 0.33	11.66 ± 0.82	30.16 ± 1.54	7.50
PAMPSAM	−0.09 ± 0.11	−0.13 ± 0.01	−0.13 ± 0.32	−0.04 ± 0.11	3.99
